# *Cryptococcus* flips its lid - membrane phospholipid asymmetry modulates antifungal drug resistance and virulence

**DOI:** 10.15698/mic2016.08.521

**Published:** 2016-08-01

**Authors:** Erika Shor, Yina Wang, David S. Perlin, Chaoyang Xue

**Affiliations:** 1 Public Health Research Institute Center, New Jersey Medical School, Rutgers University, Newark, New Jersey, USA.; 2 Department of Microbiology, Biochemistry and Molecular Genetics, New Jersey Medical School, Rutgers University, Newark, New Jersey, USA.

**Keywords:** lipid flippase, Cdc50, Cryptococcus neoformans, antifungal drug resistance, virulence

## Abstract

Human fungal infections are increasing in prevalence and acquisition of antifungal drug resistance, while our antifungal drug armamentarium remains very limited, constituting a significant public health problem. Despite the fact that prominent antifungal drugs target the fungal cell membrane, very little is known about how fungal membrane biology regulates drug-target interactions. Asymmetrical phospholipid distribution is an essential property of biological membranes, which is maintained by a group of transporters that dynamically translocate specific phospholipid groups across the membrane bilayer. Lipid flippase is the enzyme responsible for translocation of certain phospholipids, including phosphatidylserine (PS), across the plasma membrane from the exocytoplasmic to the cytoplasmic leaflet. Loss of lipid flippase leads to abnormal phospholipid distribution and impaired intracellular vesicular trafficking. The recent research article by Huang *et al.* reported that in pathogenic fungus *Cryptococcus neoformans* loss of lipid flippase activity sensitized cryptococcal cells to multiple classes of antifungal drugs, including the cell wall active echinocandins, and abolished fungal virulence in murine models. This finding demonstrates that lipid flippase may promote fungal drug resistance and virulence and indicates that this enzyme may represent a novel antifungal drug target.

Cryptococcosis, predominantly caused by *Cryptococcus neoformans*, is a deadly fungal disease that is projected to cause nearly 620,000 deaths annually. Treatment options for cryptococcosis are very limited. Traditionally used antifungal drugs are either highly toxic (polyenes), which severely limits their use, or fungistatic (triazoles), which necessitates long treatment regimens and leaves open the avenue for emergence of drug resistance.

The echinocandins are the newest antifungal drug class, which shows fungicidal activity against several major fungal pathogens, such as *Candida* and *Aspergillus*. The target of the echinocandins is β-1,3-glucan synthase, an enzyme that produces β-1,3-D-glucan, a major cell wall component. In *C. neoformans*, β-1,3-glucan synthase is encoded by a single gene, *FKS1*, which is essential for viability. Furthermore, purified cryptococcal β-glucan synthase is sensitive to echinocandin drugs *in vitro. *Nevertheless, *C. neoformans* is naturally resistant to echinocandins, which are completely ineffective in treating cryptococcosis as well as several other important mycoses, including histoplasmosis and mucormycosis. The resistance mechanism remains unknown and appears to be independent of melanin and capsule, two virulence factors unique to *Cryptococci.* Recently Huang *et al.* reported that loss of lipid flippase activity resulting from deletion of the *CDC50* gene in *C. neoformans* sensitizes this fungus to caspofungin, a drug of echinocandin class. Furthermore, the *cdc50*∆ mutant was also hyper-sensitive to triazoles and was completely avirulent in a murine model of systemic cryptococcosis. These results have implicated cryptococcal lipid flippase in drug resistance and virulence of this deadly fungal pathogen.

Lipid flippases are enzymes responsible for maintaining lipid asymmetry of cellular (both plasma and organelle) membranes whereby different leaflets of the same membrane have distinct lipid compositions. For instance, the exoplasmic leaflet of the plasma membrane is enriched in sphingolipids and phosphatidylcholine (PC), whereas the cytoplasmic leaflet is enriched in phosphatidylserine (PS) and phosphatidylethanolamine (PE). Lipid flippases catalyze the translocation of specific phospholipids across membranes to promote this asymmetry, which is essential for a number of membrane-dependent processes, including vesiculation, budding, cell signaling, and cell motility. While the catalytic flippase activity is carried out by type IV P-type ATPases (P4-ATPases), most lipid flippases also require the presence of a regulatory subunit, which is encoded by *CDC50* homologs (Fig. 1). It has been shown that Cdc50 interacts with P4-ATPases via the Cdc50 exoplasmic loop and that this interaction is essential for flippase activity. Yet, how specifically Cdc50 regulates P4-ATPase activity is still being investigated. In *C. neoformans*, there are four P4-ATPase homologs but only one Cdc50 homolog, suggesting that in this organism Cdc50 may regulate multiple P4-ATPases.

**Figure 1:**
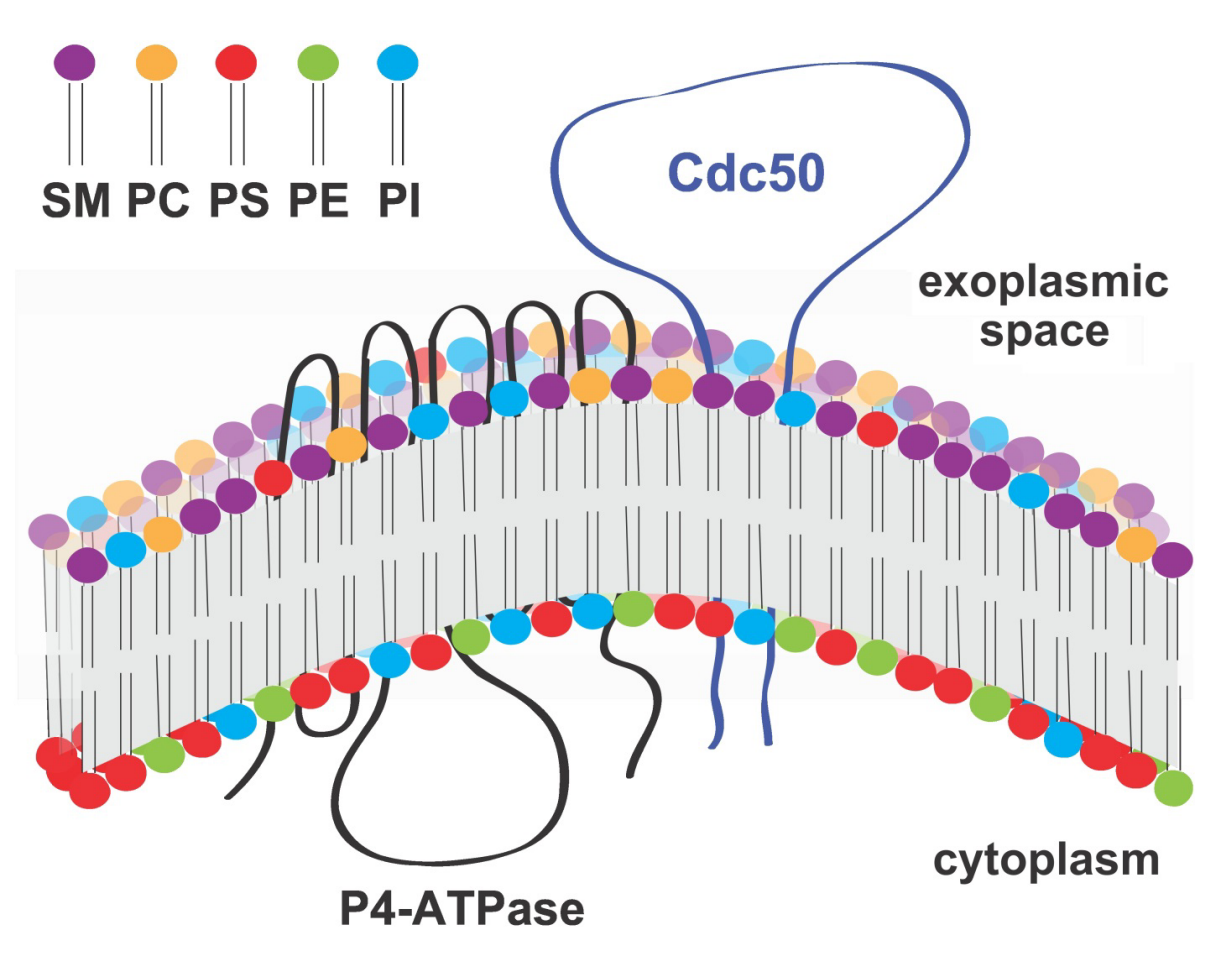
FIGURE 1: **Predicted topological model of P4-ATPase and its regulatory subunit Cdc50.** Cdc50 interacts with P4-ATPase through its exocytoplasmic loop to activate the lipid flippase function, which translocates specific phospholipids from the exocytoplasmic leaflet to the cytoplasmic leaflet of the lipid bilayer membrane. SM: sphingomyelin, PC: phosphatidylcholine, PS: phosphatidylserine, PE: phosphatidylenthanolamine, PI: phosphatidylinositol.

The *cdc50*∆ mutant strain was identified by Huang *et al.* in a screen for mutants sensitive to echinocandin drug caspofungin, and this report is the first examination of lipid flippase function in *C. neoformans*. Importantly, the authors showed that Cdc50 is essential for lipid flippase function in *C. neoformans* by demonstrating that *cdc50*∆ blocks PS import across the plasma membrane. Additionally, using a fluorescently labeled caspofungin, the authors demonstrated that the *cdc50*∆ mutant binds and takes up caspofungin better than the wild type strain, which may explain the drug sensitivity phenotype. Interestingly, the *cdc50*∆ mutant also showed increased sensitivity to other drug classes, including triazoles fluconazole and voricanzole and polyene drug amphotericin B. However, the *cdc50*∆ mutant remained highly resistant to two other drugs of the echinocandin class, micafungin and anidulafungin, suggesting that specific drug-fungal cell interactions underlie the observed drug sensitivity effects and arguing against the notion that loss of lipid flippase causes a general membrane permeability defect.

How the lipid flippase influences drug-fungal cell interactions remains to be understood. Currently, two non-mutually exclusive hypotheses exist (Fig. 2). In the first model, increased levels of PS (or other phospholipids) on the outer leaflet of the plasma membrane in the *cdc50*Δ mutant may change membrane structure in a way that enhances the interaction of certain drugs with their targets. In an alternative model, because lipid flippase activity is required for normal intracellular vesicle trafficking, the *cdc50*Δ mutant may cause incorrect trafficking of vesicles containing specific drug targets (i.e. β-1,3-D-glucan synthase for echinocandins, ergosterol for polyenes, and lanosterol 14-α-demethylase for triazoles), impairing these pathways and increasing sensitivity to the corresponding antifungal drugs. The first hypothesis is feasible because phospholipid distribution of membranes is a critical determinant of membrane structure and protein content. The second hypothesis is supported by recent reports showing that in *Saccharomyces cerevisiae* a lipid flippase mutant caused the mis-sorting of membrane proteins and ergosterol: instead of being trafficked to the plasma membrane via the late ER-Golgi network, membrane proteins and ergosterol were shuttled to the vacuole. However, this scenario does not explain why the *cdc50*∆ mutant differentially affects sensitivity to different echinocandin drugs; thus, if this model is correct, it may operate together with other mechanisms, e.g. model 1 (Fig. 2).

**Figure 2:**
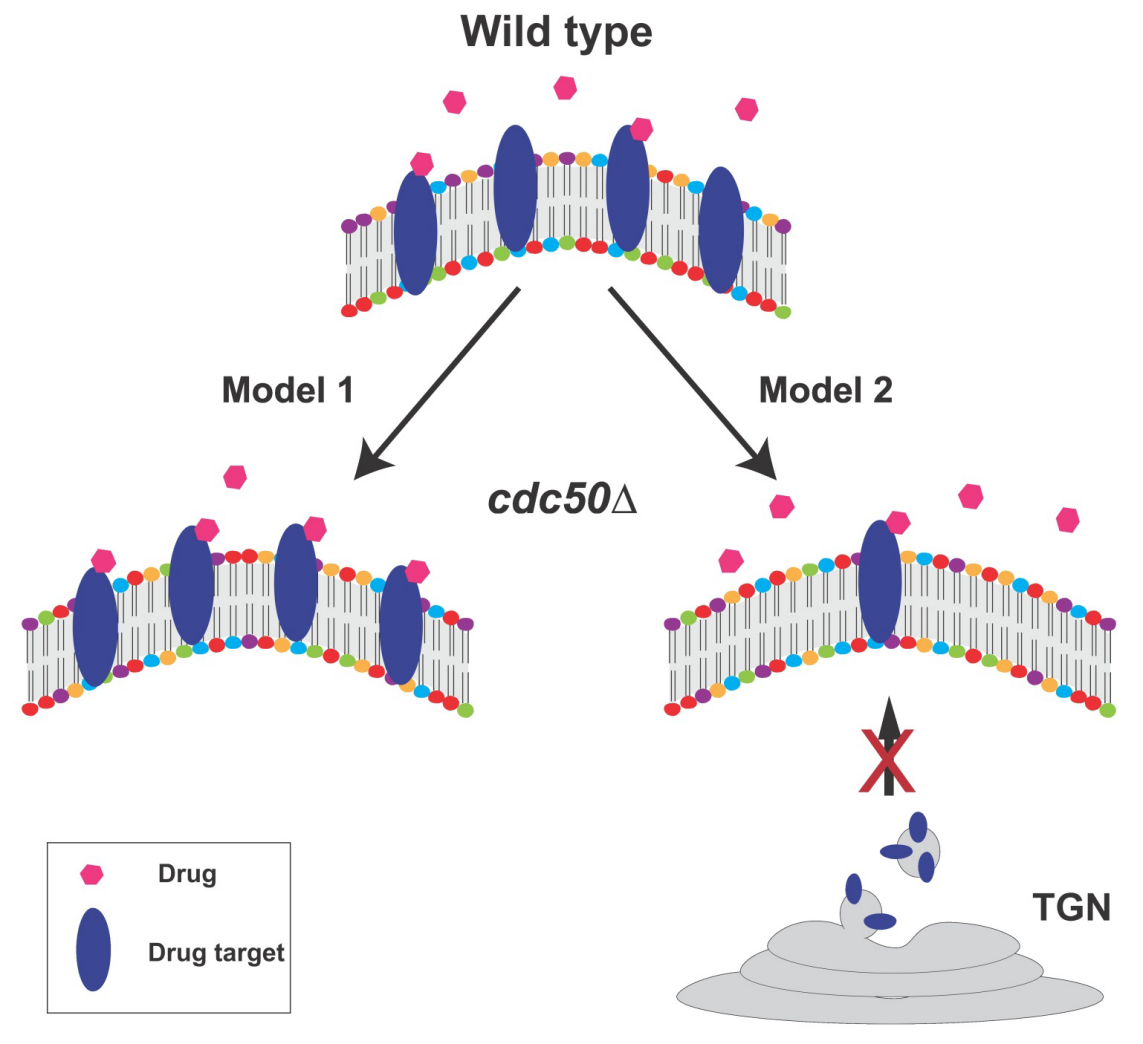
FIGURE 2: **Two alternative, non-mutually exclusive working models of lipid flippase function in antifungal drug resistance.** In Model 1, increased levels of PS (or other phospholipids) on the outer leaflet of the plasma membrane in the *cdc50*∆ mutant may change membrane structure in a way that promotes the interaction of certain drugs with their targets, thus enhancing drug-mediated inhibition of target activity. In Model 2, because lipid flippase activity is required for normal intracellular vesicle trafficking, vesicles containing specific drug targets may be incorrectly targeted, reducing correct drug target localization, impairing these pathways, and increasing sensitivity to the corresponding antifungal drugs. TGN: trans-Golgi network.

To test the first hypothesis, one can determine the impact of PS exposure on cryptococcal drug resistance by analyzing other mutants with altered surface PS levels, e.g. lipid scramblase (less surface PS), PS synthase (no PS production), and lipid flippase P4-ATPase subunits (increased surface PS). Alternatively, it is possible to increase surface PS levels using chemical induction, e.g. calcium ionophore. To test the second hypothesis, one can track the intracellular trafficking and distribution of fluorescently-labeled drug targets in wild type and *cdc50*∆ cells. Finally, it would also be valuable to utilize the recently developed lipidomics and proteomics approaches to analyze the overall impact of lipid flippase on membrane composition and structure, which could lead to a better understanding of the role of lipid flippase in antifungal drug resistance.

Huang *et al.* also demonstrated that lipid flippase plays an important role in development of cryptococcosis in a murine model: the *cdc50*∆ mutant was avirulent and was completely cleared in the mouse lung after 7 days post-infection. Interestingly, the loss of fungal virulence in the mutant appeared to be independent of classical cryptococcal virulence factors, such as growth at 37°C, melanin and capsule. Indeed, melanin production was not affected in the *cdc50*∆ mutant, and the mutant cells only had a very modest slow growth phenotype at 37°C. Additionally, the mutant produced an even larger capsule than the wild type strain. This is a surprising feature of an avirulent strain because polysaccharide capsule has been recognized as an important disease mechanism that has anti-inflammatory properties and acts to suppress host immunity. Whether the enlarged capsule plays a role in drug interactions or the avirulent phenotype of the *cdc50*∆ is still unclear.

The authors presented a hypothesis to explain the avirulence phenotype of the *cdc50*∆ mutant by focusing on fungus-macrophage interactions. Alveolar macrophages are the first line of host defense against cryptococcal infection, and *C. neoformans* is a facultative intracellular pathogen that can survive inside macrophages under certain conditions. It has been well established that increased PS exposure on the mammalian cell surface (e.g. on apoptotic cells) acts as a signal for macrophage recognition and phagocytosis. Thus, the authors tested the hypothesis that loss of Cdc50 leads to increased phagocytosis of the fungal cells. Indeed, using a macrophage cell line they found that the *cdc50*∆ strain was engulfed and killed more efficiently than the wild type strain. How increased PS exposure on the plasma membrane generates a signal that is recognized by host macrophages despite the presence of the fungal capsule and thick cell wall is still unclear. It is possible that macrophages may secrete factors that survey the environment and help recognize potential target cells for phagocytosis.

Despite the fact that almost all existing antifungal drugs target the fungal cell surface (membrane or cell wall), very little is known about how fungal membrane biology regulates drug-target interactions. For example, how phospholipid composition and distribution contribute to fungal drug interactions and uptake has been entirely unexplored. The discovery that lipid flippase helps *C*. *neoformans* resist common antifungal drugs and is essential for fungal virulence underscores the importance of investigating these questions and highlights the potential of lipid flippase as a new antifungal drug target.

